# Allochthonous Carbon—a Major Driver of Bacterioplankton Production in the Subarctic Northern Baltic Sea

**DOI:** 10.1007/s00248-015-0714-4

**Published:** 2015-12-17

**Authors:** D. Figueroa, O. F. Rowe, J. Paczkowska, C. Legrand, A. Andersson

**Affiliations:** Department of Ecology and Environmental Science, Umeå University, SE-901 87 Umeå, Sweden; Umeå Marine Sciences Centre, SE-905 71 Hörnefors, Sweden; Ecology and Evolution in Microbial Model Systems, EEMiS, Department of Biology and Environmental Sciences, Linnaeus University, SE-391 82 Kalmar, Sweden; Department of Food and Environmental Sciences, Division of Microbiology and Biotechnology, Viikki Biocenter 1, University of Helsinki, Helsinki, Finland

**Keywords:** Allochthonous organic matter, Carbon utilization, Bacterioplankton production, Sub-arctic estuary, Baltic Sea

## Abstract

Heterotrophic bacteria are, in many aquatic systems, reliant on autochthonous organic carbon as their energy source. One exception is low-productive humic lakes, where allochthonous dissolved organic matter (ADOM) is the major driver. We hypothesized that bacterial production (BP) is similarly regulated in subarctic estuaries that receive large amounts of riverine material. BP and potential explanatory factors were measured during May–August 2011 in the subarctic Råne Estuary, northern Sweden. The highest BP was observed in spring, concomitant with the spring river-flush and the lowest rates occurred during summer when primary production (PP) peaked. PLS correlations showed that ∼60 % of the BP variation was explained by different ADOM components, measured as humic substances, dissolved organic carbon (DOC) and coloured dissolved organic matter (CDOM). On average, BP was threefold higher than PP. The bioavailability of allochthonous dissolved organic carbon (ADOC) exhibited large spatial and temporal variation; however, the average value was low, ∼2 %. Bioassay analysis showed that BP in the near-shore area was potentially carbon limited early in the season, while BP at seaward stations was more commonly limited by nitrogen-phosphorus. Nevertheless, the bioassay indicated that ADOC could contribute significantly to the in situ BP, ∼60 %. We conclude that ADOM is a regulator of BP in the studied estuary. Thus, projected climate-induced increases in river discharge suggest that BP will increase in subarctic coastal areas during the coming century.

**Electronic supplementary material:**

The online version of this article (doi:10.1007/s00248-015-0714-4) contains supplementary material, which is available to authorized users.

## Introduction

Autochthonously produced organic carbon has been shown to be a major driver of bacterial production (BP) in many aquatic systems [16]. However, in systems greatly influenced by allochthonous organic carbon, such as low-productive humic lakes, bacteria are decoupled from autotrophic phytoplankton due to the high availability of allochthonous dissolved organic matter (ADOM) [38]. Subarctic estuaries are also highly influenced by ADOM, especially during the spring river-flush [50]. The terrestrial inflow of organic matter may thus promote BP, since the availability of food substrates is often a growth-limiting factor in natural aquatic systems [17, 30, 61]. However, BP may also be driven by autochthonous production. Presently, it is poorly understood whether BP in subarctic estuaries is mostly driven by autochthonous organic carbon or by river-discharged ADOM.

Dissolved organic carbon (DOC) in general makes up a large part, ∼50 %, of the reduced dissolved organic matter (DOM) in aquatic ecosystems [21]. Some ADOM entering coastal systems forms into aggregates which sink to the benthic system, whilst the rest remains in dissolved form in the water [32]. ADOM consists of different fractions, which have varying properties. Humic substances, which are composed of both fulvic and humic acids, represent a significant part of the river-borne allochthonous DOC (ADOC) [11]. ADOM is relatively refractory and contains compounds that absorb light, i.e. chromophoric dissolved organic matter (CDOM), thus reducing light penetration in the water column which may cause decreasing phytoplankton production [4, 13, 32].

The ecological and biogeochemical significance of DOM is, to a high degree, linked to the key role of bacteria in carbon (C) and nutrient cycling [16, 35]. DOM constitutes a reservoir of reduced carbon and essential nutrients, such as nitrogen (N) and phosphorous (P), and its biological degradation is influenced by environmental factors such as temperature or nutrient limitation [31, 40]. Only a small fraction of ADOM is available as a bacterial carbon substrate [56], while higher proportions of the ADOM-bound N and P have been shown to be available for bacterial growth [53]. Bacteria either transform the DOM into biomass, which can then transfer through the food web to higher trophic levels, or use the carbon for their own metabolism and respiration [18]. The bacterioplankton production and the community composition are also influenced by the composition of the DOM, since different bacterial groups have varying capacities to metabolize organic substances in the DOM pool [24, 42, 49].

In areas influenced by freshwater discharge, such as estuarine systems in the Baltic Sea, ADOM is likely to affect the productivity and trophic balance of the ecosystem [5, 58]. Heterotrophic bacteria may be selectively promoted by ADOM inputs; however, the bacterial growth response will depend on the bioavailability of carbon and nutrient content in the ADOM, as well as the growth-limiting substance for heterotrophic bacteria. In coastal areas of the Baltic Sea, bacterioplankton production has been shown to be limited by organic carbon or by nutrients [30, 49]. The variations may be due to different composition and quality of the organic substances, as well as the different substrate requirements of bacteria [11, 50]. Furthermore, seasonality and the flows or pulses from rivers can have an important impact on heterotrophic carbon consumption in coastal estuaries since the DOM quality and concentration vary depending on surrounding terrestrial and hydrological processes [29].

The distribution, timing and concentration of discharges from terrestrial to aquatic ecosystems are expected to vary in subarctic boreal areas due to climate change [28]. In the Bothnian Bay, the northernmost part of the Baltic Sea, modifications in the annual timing of snow melt, river flow patterns and nutrient discharges are expected [25, 28, 34]. Consequently, the transport of inorganic substances and ADOM will change [54], providing bacteria with pulses and extended periods of elevated potential carbon and nutrient sources and reducing the available light for phytoplankton production in the water column [4, 12, 13, 52]. Consequently, the structure and function of phytoplankton and bacterial communities will be altered, resulting in a modified basal production balance [4, 52]. Such alterations at the seasonal level, and certainly over extended periods (e.g. long term perspectives), could modify the structure, function and productivity of the whole food web [12, 16, 52].

The objective of this study was to elucidate which factors drive BP in subarctic estuaries influenced by high concentrations of ADOM. Our hypotheses were the following: (1) In subarctic estuaries, BP is mainly governed by ADOM; (2) bacterial uptake of ADOM will vary over time and space, with high BP in spring at the river station due to elevated concentrations of fresh ADOM; and (3) although high ADOC loads occurs in subarctic estuaries, BP can potentially be carbon limited due to the simultaneous supply of highly available NP in the river-borne DOM. The Råne Estuary, situated at the northernmost extreme of the Baltic Sea, served as the study system. This sea area is highly influenced by river inflow and as much as ∼87 % of the DOM is of terrestrial origin [2]. Our approach combined field studies and bioassays. The results are discussed from a climate change perspective, since it has been predicted that precipitation and river inflow will increase in such areas during the coming century [5].

## Methods

### Study Site

**Fig. 1:**
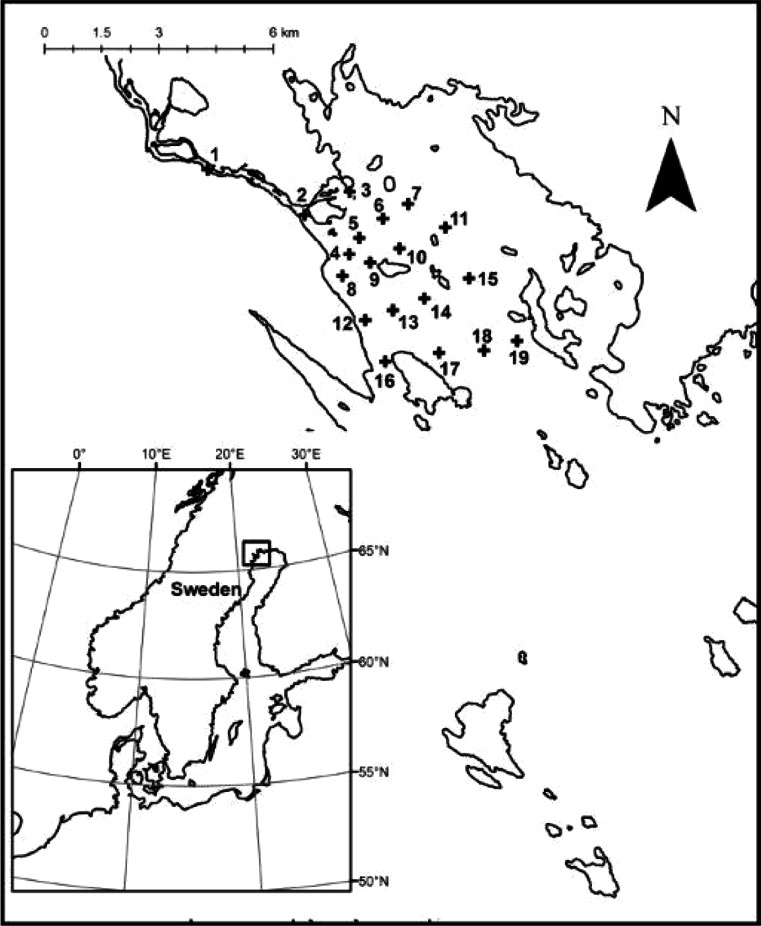
Position of sampling stations in the Råne River and Estuary, Bothnian Bay (northern Baltic Sea)

Sampling was performed in the Råne Estuary (the Bothnian Bay, Baltic Sea, Fig. 1). The estuary is subject to river discharge (Table 1) from an unregulated river (the Råne River) running through a largely forested catchment area in northern Sweden.

**Table 1:** Average values (and range) of physicochemical variables at 19 stations in the Råne River and Estuary during the 2011 productive season

	May	June	July	August
Date	10–12	20–22	11–13	9–11
DOC (mg l^−1^)	7.6 (5.0–12.8)	5.6 (4.4–6.9)	6.3 (5.2–7.3)	5.9 (4.7–7.3)
Humic substances (μg l^−1^)	61.5 (39.9–67.6)	43.8 (25.9–68)	53.6 (32.7–71.4)	41.9 (23.1–63.1)
SPM organic (g m^−3^)	1.5 (0.4–2.5)	1.4 (0.8–2.4)	1.5 (1,0–2.4)	1.4 (0.8–2.5)
CDOM (m^−1^)	3 (1.5–4.2)	2.8 (1.8–5.5)	2.9 (1.8–4.2)	2.1 (1.1–3.7)
TotP (mg l^−1^)	0.009 (0.008–0.01)	0.01 (0.006–0.016)	0.01 (0.008–0.014)	0.01 (0.008–0.016)
TotN (mg l^−1^)	0.38 (0.29–0.46)	0.28 (0.23–0.37)	0.29 (0.23–0.41)	0.29 (0.24–0.36)
Temperature (°C)	6.7 (5.8–8)	15.7 (14.1–16.6)	21.4 (19.8–23.0)	16.5 (15.7–17.4)
pH	6.9 (6.6–7.1)	7.2 (6.6–7.6)	7.2(6.8–7.4)	7.4 (7.0–7.6)
Salinity units	0.3 (0–1.1)	0.6 (0–1.7)	0.5 (0–1.1)	1.0 (0–1.9)
River discharge (m^3^ s^−1^)	99.3 (96–103)	33.4 (32.1–35.1)	33.1 (31.8–34.5)	24.6 (23.8–26.0)

Mean and range are for a single given sampling month, encompassing all 19 sampled stations (*n* = 19)

### Field Sampling

Physicochemical and biological variables were measured at 19 stations in the Råne River-Estuary (Fig. 1S, Table 1). One station was situated in the river and constituted the source sampling point, and the other 18 stations were located within the estuary (referred to here-after as the seaward stations). The stations were distributed over a 16 km^2^ area (Fig. 1) and were sampled monthly from May to August 2011. The first sampling was undertaken immediately following the inland snow-melt and the last towards the end of the productive season (Table 1). Each sampling occasion spanned a 3-day period. Water was collected at a depth of 1 m using a Ruttner sampler. Samples were analysed or preserved within four hours of collection.

### Physicochemical Variables

Temperature was measured in situ. pH and conductivity were measured in the laboratory at 25 °C (Mettler Toledo) and converted to the actual values for the in situ temperature according to Fofonoff and Millard [23]. Total phosphorus (TotP) and total nitrogen (TotN) were analysed using a Bran & Luebbe TRAACS 800 autoanalyser according to Grasshof et al. [26].

A number of variables indicative of ADOM were measured, including dissolved organic carbon (DOC), chromophoric dissolved organic matter (CDOM) and humic substances. Samples for DOC were filtered (0.2 μm Supor Membrane Syringe Filter, non-pyrogenic; Acrodisc), acidified with 0.1 ml of 1.2 M HCL, sparged and analysed using a Shimadzu TOC-5000. For CDOM measurement, water was filtered through an acid-washed 0.2-μm membrane filter, and the filtrate was measured spectrophotometrically (300–800 nm), as described in Kratzer et al. [41]. The concentration of humic substances was measured from whole water samples using a PerkinElmer LS 30 fluorometer (350 nm exCitation wavelength and 450 nm emission wavelength). Sulfuric acid (0.05 M) was used as a blank, and calibration standards were prepared from quinine dihydrogen sulfate dihydrate in 0.05 M sulfuric acid [14, 57]. Suspended particulate material (SPM) was estimated using the gravimetric method [55], slightly modified by filtering triplicate 1 l volumes of seawater onto precombusted (450 °C) 47 mm GFF filters. Differences in the dry weight of filters before and after the filtration represent the total SPM. Filters were then re-combusted (450 °C) to burn off the particulate organic substances. The particulate organic fraction (SPM_organic_) and the particulate inorganic fraction (SPM_inorganic_) were calculated from these weights. Since this study focuses on the bacterial utilization of organic compounds, we considered only the SPM_organic_.

### Bacterial and Primary Production

BP was measured using the ^3^H-thymidine incorporation technique as described in Berglund et al*.* [12]. One millilitre of seawater was added to three Eppendorf tubes, one control and duplicate samples. Bacteria in the control were pre-killed by adding 100 μl ice-cold 50 % TCA and incubating at −20 ^o^C for 5 min. Next, 2 μl [^3^H]-thymidine (84 Ci mmol^−1^; PerkinElmer, Massachusetts, USA) was added to each tube to a final concentration of 24 nM. The incorporated thymidine was converted to cell production using the conversion factor of 1.4 × 10^18^ cells mol^−1^ [60]. Carbon biomass production was estimated from cell production and average cell carbon biomass as described in Eriksson-Wiklund et al. [22]. Primary production (PP) was measured in situ using the ^14^C technique: 5 ml seawater was added to three 20 ml transparent polycarbonate tubes with one dark tube as a control. Next, 7.2 μl ^14^C was added to each tube (^14^C Centralen Denmark, activity 100 μCi/ml) and were incubated at 1 m depth for ∼3 h. The samples were analysed in a Beckman 6500 scintillation counter. Daily PP was calculated as described in Andersson et al. [3].

### ADOC Availability for Bacterial Growth

Temporal and spatial DOC availability was measured in a bio-assay. On each of the monthly sampling occasions, water was collected at stations 1, 2, 6, 10, 17 and 19, forming a seaward transect.

Collected water was divided into two fractions: (1) un-filtered and (2) ∼0.7 μm filtered (<15 kPa) through a pre-combusted GF/F filter (Whatman). Fractions were stored in darkness at 4 °C whilst awaiting the experiment (<48 h). At the start of the experiment, 100 ml of the un-filtered inoculum water and 900 ml of the GFF-filtered water were mixed to create the starting matrix at each individual station. For each station, 150 ml of this matrix was then distributed into six individual 200 ml cell culture flasks (polystyrene culture flask, non-pyrogenic and non-cytotoxic; Sarstedt, USA). Potential nutrient limitation was excluded by adding DIN (NH_4_Cl and NaNO_3_ at a final concentration of 0.172 and 1.06 μmol l^−1^, respectively) and DIP (KH_2_PO_4_ final concentration of 0.077 μmol l^−1^) to three of the six flasks per station. The experimental flasks, three controls (no addition) and three nutrient amended (per station), were incubated in darkness at in situ temperature for 10 days. Incubation temperature was set to the mean temperature of the sampled stations at each monthly sampling occasion (Table 1). Nutrient limitation of bacteria was determined by comparing cultures with and without nutrient addition, where the increase of BP in nutrient amended cultures (NP) showed a limitation of nutrients.

BP and DOC concentrations were measured on days 0, 2, 6 and 10 using the methods described above.

DOC availability was calculated as follows:

$$ {\mathrm{DOC}}_{\mathrm{consumed}}\left(\%\right)=\left({\mathrm{DOC}}_0\hbox{-} {\mathrm{DOC}}_{\mathrm{X}}\right)\ /\ {\mathrm{DOC}}_0\times 100 $$

where DOC_0_ and DOC_X_ are the DOC concentrations at day 0, and on the last day that bacteria were growing in the cultures. In May, this sampling was between day 0 and day 6, while in June, July and August it was between day 0 and day 2.

Bacterial growth efficiency (BGE) was calculated by dividing the total bacterial carbon production by the consumption of DOC during the growth period, as follows:

$$ \mathrm{B}\mathrm{G}\mathrm{E}\ \left(\%\right) = {\mathrm{BP}}_{\mathrm{int}\ \mathrm{day}\ 0-\mathrm{X}}/\ \left({\mathrm{DOC}}_0\hbox{-} {\mathrm{DOC}}_{\mathrm{X}}\right) $$

where BP_int_ is the integrated BP and the subscript numbers (for both BP and DOC) denote the active growth phase. For the May sampling, this period was from days 0 to 6, and for the June, July and August samplings, from day 0 to day 2.

The proportion of BP that was potentially based on in situ concentrations of DOC was calculated according to the equation:

$$ {{\mathrm{BP}}_{\mathrm{DOC}}}_{in\  situ}\left(\%\right)=\mathrm{B}\mathrm{G}\mathrm{E} \ast \left({\mathrm{DOC}}_{in\  situ} \ast {\mathrm{DOC}}_{\mathrm{consumed}}/\ {\mathrm{Days}}_{\mathrm{ADOC}\ \mathrm{usage}}\right)/\ {\mathrm{BP}}_{in\  situ} $$

In this calculation, we assume that the major part of the isolated DOC is of allochthonous origin [19].

### Statistical Analyses

Partial least square (PLS) analysis was used to elucidate the combined effects of physicochemical and biological variables on BP (SIMCA version 13.0.3). The results are presented as a principal component graph with the monthly sampling events at each station distributed depending on the influence of each variable. Kendall-Tau correlations were used to investigate the relationship between river discharge and carbon concentration in the water (SPSS Statistics 22). The individual influence of each variable on BP was analysed using Spearman correlation (R Studio 2.13.2). Potential nutrient (NP) limitation of bacterial metabolism in the DOC consumption bioassay was examined using linear mixed models (R Studio 2.13.2, package “lme4” version 0.999375-42), comparing controls and cultures with nutrient addition. Significant differences (*ρ* < 0.05) were considered indicative of nutrient limitation (NP).

## Results and Discussion

### River Inflow Influence on Estuarine ADOM Concentrations

The physicochemical conditions in the Råne estuary exhibited large temporal and spatial variations (Table 1). In May, the spring river flush caused an increase in concentrations of ADOM-related variables: DOC (Kendall-Tau *b* test_(two tailed)_ = 0.261, **p < 0.01), humic substances (Kendall-Tau b test_(two tailed)_ = 0.333, **p < 0.01) and CDOM (Kendall-Tau *b* test_(two-tailed)_ = 0.318, **p < 0.01). On later sampling dates, the mean concentration of ADOM variables decreased (Table 1). Large differences in concentrations were observed between the river sampling point (station 1) and those stations more distant from the river (the more seaward stations). This trend was most distinct in spring, when large river discharges occurred, as compared with late summer, for example, as much as a ∼60 % difference in DOC concentration was recorded between the river (12.8 mg l^−1^) and seaward (5 mg l^−1^) stations in May, while the difference was only 25 % in August (6.5 mg l^−1^ at station 3 and 4.7 mg l^−1^ at more seaward stations). Similar trends were observed for humic substances and TotN (Table 1 and Fig. 4b). TotP concentrations were markedly lower than TotN concentrations (Table 1), no increase was recorded due to elevated river flow in May and mean concentrations remained similar at all sampling events (Table 1 and Fig. 4c). The SPM_organic_ and CDOM were relatively stable across the estuary and throughout the season; however, CDOM decreased slightly in August (Table 1). High river discharge was associated with lower pH in the estuary, especially in May (Kendall-Tau *b* test_(two-tailed)_ = 0.505, **p < 0.01, Table 1), probably as a consequence of high concentrations of ADOM, containing compounds such as fulvic and humic acids [43]. Temperature showed larger temporal than spatial variation, with the lowest values in May and highest in July (Table 1). Taken together, freshwater inputs were found to have a large influence on the concentrations of ADOM variables in the estuary, especially during the elevated river discharge in May (Table 1).

### Relationship Between Bacterial Production and ADOM

**Fig. 2:**
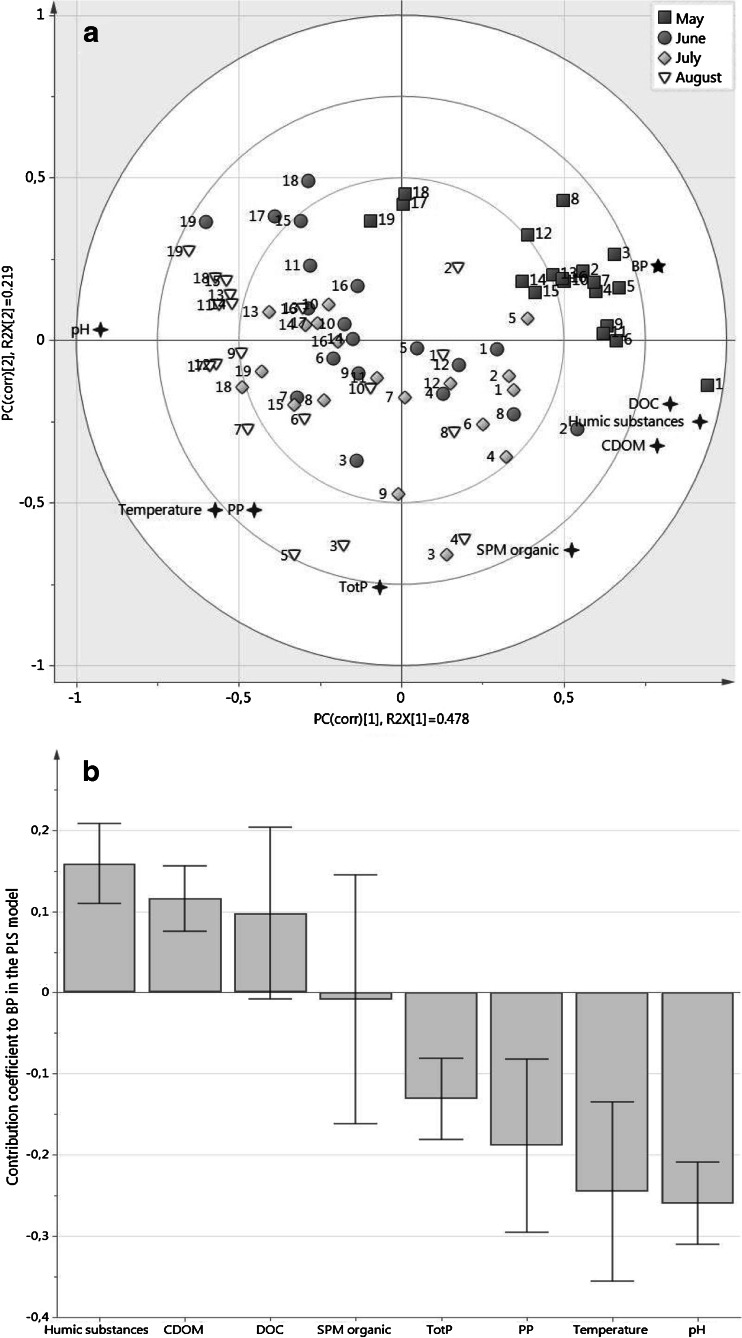
Partial least square regression (PLS) model: (**a**) PLS biplot projection on the influence of humic substances, dissolved organic carbon (DOC), suspended particulate organic matter (SPM org), total phosphorous concentration (TotP), temperature (temp), chromophoric dissolved organic matter (CDOM), pH and primary production (PP) on the bacterial production (BP) in the Råne estuary. *R* ^2^ *X* is the cumulative information contained on each axis. **b** Regression coefficients plot showing the degree of influence of the environmental variables during the season on BP in the PLS model. The analysis was based on sampling at 19 stations, four times during the period May–August 2011

ADOM influenced the pelagic BP, as indicated by the PLS model (Fig. 2). The PLS projection showed a two-component distribution containing 67.8 % of the cumulative information from the original data. Cross-validation analysis of the PLS model revealed a good fit with measured data (ANOVA: *p* = 2.7 × 10^−15^***). The ADOM components (DOC, humic substances and CDOM) were situated close to BP in the PLS projection (Fig. 2a) and were positively correlated with BP (PLS *ρ*_combined_ = 0.67, 0.55 and 0.58, respectively, Table 2). Organic SPM was poorly correlated to BP (Table 2) and seemed to exert little influence (Fig. 2b). Temperature, pH and TotP were negatively correlated with BP (Table 2 and Fig. 2b). These variables were instead positively correlated with PP (*p* = 0.004, 0.0006 and 0.052 respectively), which itself was placed opposite BP in the PLS projection, and negatively correlated, *ρ*_combined_ = −0.44 (Fig. 2b).

**Table 2:** Influence of environmental variables (physicochemical and biological) on bacterial production in the Råne Estuary

Variables	PLS correlation coefficient (*ρ*_combined_)	Spearman’ s correlation coefficient (*ρ*)
Humic substances	0.67	0.70***
Chromophoric dissolved organic carbon (CDOM)	0.55	0.63***
Total nitrogen concentration (TotN)	– –	0.65***
Dissolved organic carbon (DOC)	0.58	0.64***
Organic suspended particular material (SPM organic)	0.28	0.36**
Total phosphorous concentration (TotP)	−0.18	0.09
Temperature	−0.57	−0.43***
Primary production (PP)	−0.44	−0.52***
Salinity	– –	−0.62***
pH	−0.78	−0.80***

Correlation coefficients of the combined effects on bacterial production, calculated using a PLS correlation model (*ρ*_combined_) and the individual environmental factor effect on bacterial production, calculated using Spearman’s correlations (*ρ*)

The *p* values for the Spearman’s correlation were obtained using a two-tailed *t* test, with level of significance indicated by *p < 0.05, **p < 0.01, ***p < 0.001. Environmental variables excluded from the PLS model are marked with “– –” in the table

**Fig. 3:**
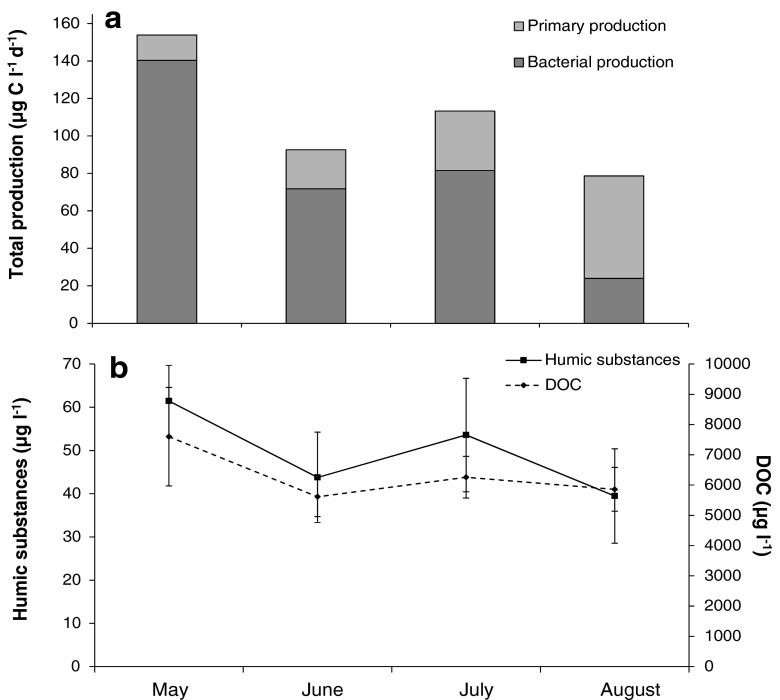
Average BP and PP (**a**), humic substances and DOC concentrations (**b**) in surface waters in the Råne Estuary during the period May–August 2011. *Error bars* denote ±1 standard deviation

**Fig. 4:**
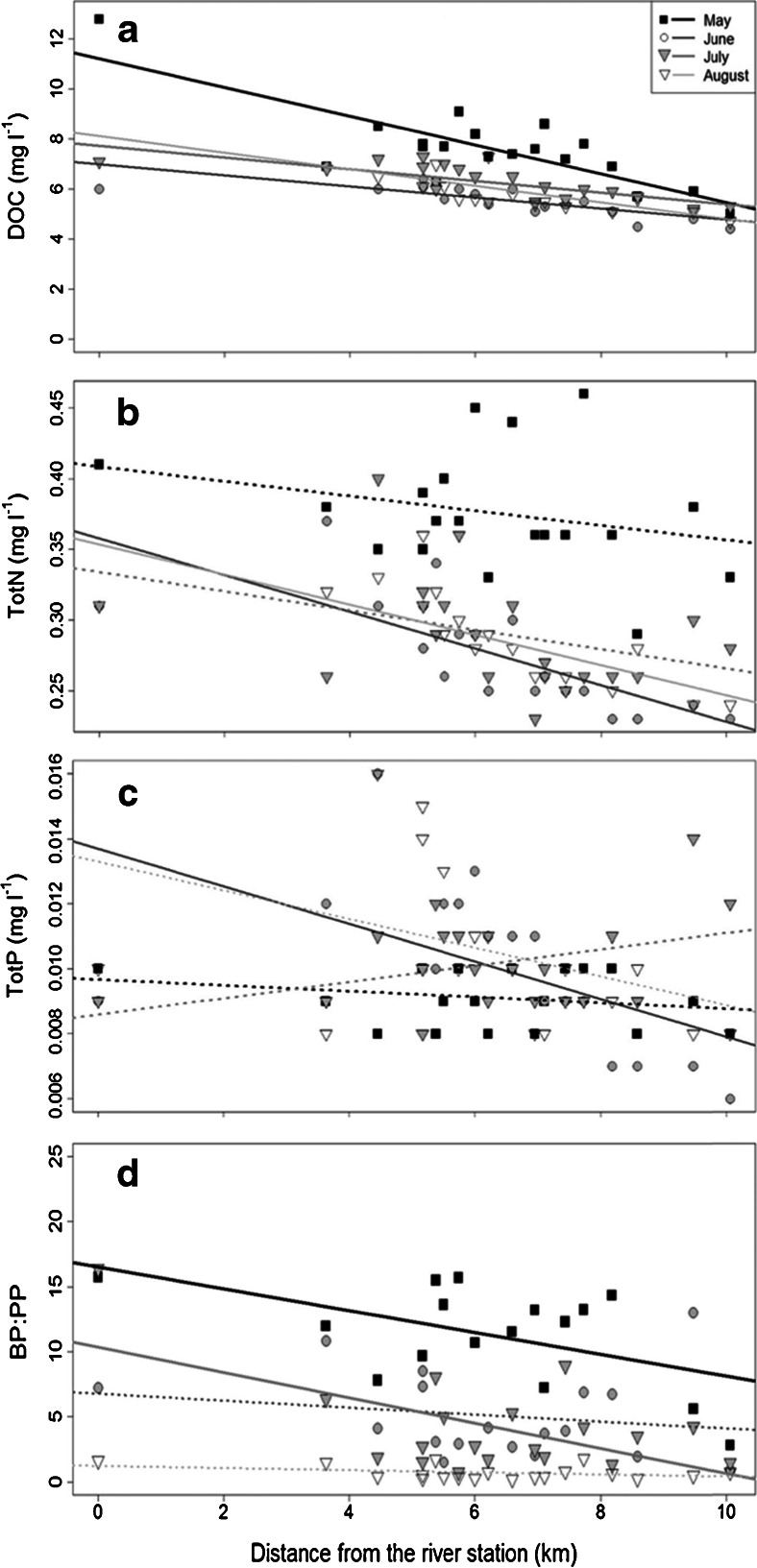
Concentrations of DOC (**a**), TotN (**b**), TotP (**c**) and the BP/PP ratio (**d**) along a river-sea gradient during different months (May–August). *Solid lines* show significant upwards or downwards trends along the transect (*p* < 0.05) and *dashed lines* indicate non-significant trends

The large majority of the DOC in the studied sea area is of terrestrial origin [2], and the data support our hypothesis that ADOM controls pelagic BP in this subarctic estuary. BP followed the same temporal pattern as DOC and humic substances (Fig. 3a, b), being highest in May and decreasing with declining river discharge (Table 1). The DOC concentration decreased from 7.6 mg l^−1^ in May to ∼5.9 mg l^−1^ in August, while humic substances, though relatively low in concentration, also decreased from ∼62 to ∼40 μg l^−1^ in August when the river discharges were low. However, PP showed the opposite trend to DOC and humic substances, being lowest in May and highest in August (Fig. 3a). The dominance of BP during the study period, constituting on average 73 % of the basal pelagic production, indicates that the Råne Estuary is a net heterotrophic system (Fig. 3a, d). This dominance of BP could, in principle, be due to both bottom-up and top-down factors, but resource limitation of BP has been shown to overshadow predation-induced limitation in the northern Baltic Sea [51]. Riverine discharge could have controlled the balance of autotrophic (phytoplankton) and heterotrophic (bacterial) production, either directly or indirectly. The brown colour of the river water caused shading in the estuarine water, which may have negatively affected the phytoplankton PP. This assumption is supported by the fact that in May and June, when the ADOM river discharge was highest, the BP/PP ratio was higher close to the river than at the more seaward stations (Fig. 4d). In conjunction with the increase in ADOM in the system, it appears that BP is decoupled from PP in the studied estuary.

Many studies have shown that the bioavailability of bulk DOC is low, 14–19 % [44, 56], while autochthonously produced DOC (e.g. sugars, amino acids and proteins) is rapidly consumed by bacteria. Bacteria consuming autochthonous DOC produce relatively large biomasses and have low metabolic costs [11]. Since ADOC generally consists of large and recalcitrant molecules, extracellular enzymatic degradation is needed, increasing the respiration rate and thus losses of carbon as CO_2_ [18]. Nevertheless, where bioavailable, terrestrial DOC can be an important potential growth substrate for bacteria. Cole and Caraco [15] reported than as much as 70 % of bacterial respiration was supported by old ADOM when it was transported along the Hudson River to the sea. Similar results have been reported in different aquatic ecosystems, for example in low-productive lakes [42], in subarctic and alpine lakes in Sweden [39] and in estuaries in Australia [29]. Likewise, Karlsson et al. [39] showed some seasonal effects when bacterioplankton was supported by ADOC, with high bacterial activity reported in spring directly after snow-melt, at peak ADOC concentrations. Similarly, the highest bacterial activity was recorded during early spring in the Råne Estuary (Fig. 3), when DOC concentrations were elevated (12.8 mg/l in the river and 5.0–9.1 mg/l in the estuary). Moreover, BP was positively correlated with DOC (*ρ* = 0.64) and strongly correlated with humic substances (*ρ* = 0.70), suggesting that DOC of terrestrial origin could be used as a carbon and energy source by bacteria, especially in spring when this resource was abundant (Table 2 and Fig. 3).

Although not measured, respiration would have occurred during our sampling, releasing carbon to the atmosphere in the form of carbon dioxide (CO_2_). In the Råne Estuary, most of the DOC is of terrestrial origin and contains recalcitrant compounds [2, 7]. Bacterial degradation of such substrates would increase the respiration, and the consequent release of CO_2_ in coastal areas could be of significance for biogeochemical cycles. However, ADOC can also be the main source fuelling bacterial growth, even if a large part of the carbon is lost as CO_2_ [11, 52]. Different bacterial communities are acclimated to utilize specific substrates [24], and we find it reasonable to assume that the in situ bacterial community was adapted to degrade and utilize terrestrial ADOC as a food source. The Gulf of Bothnia is a low-productive ecosystem, where BP currently serves as a fundamental productive component at the base of the pelagic food web [11, 52]. Our results suggest that an increase in ADOM concentration, as predicted in regional climate models [5], will probably further promote bacteria at the base of the trophic web. This in turn will lead to more heterotrophic-based production, which is less efficient than phytoplankton-based production for the transfer of energy up the food web [35]. The trophic balance between autotrophic and heterotrophic production may thus have implications for the total ecosystem production, including the basal level as well as intermediate and higher trophic levels [12].

A general decrease in DOC and TotN from the river to the seaward stations was recorded on all sampling occasions (Fig. 4a, b). In boreal areas, large amounts of ADOC, with different levels of reactivity, are transported from the land to the sea [50]. Various physicochemical and biological processes influence the ADOM pool, and river mouths where freshwater, replete with ADOM, mixes with marine waters have been suggested as important transformation zones [45]. Processes such as chelation can result in sedimentation of DOM as chemical complexes [46], while molecular tension produced by salinity changes and photo-degradation can make the DOM more available for bacterial consumption [40, 48, 59]. It is, however, clear that bacterial degradation of ADOM can also be significant in coastal estuaries [10, 29]. Our data generally support the suggestions that estuaries are important processing zones for ADOM; however, clear spatial and temporal variation was observed. The influence of ADOM components on BP varied over time (sampling month in Fig. 2a) and space (station distribution in Fig. 2a). BP was positively influenced by humic substances, DOC, and CDOM in the estuary in May, except for the furthest seaward stations 17, 18 and 19 (Fig. 2a). In June and July, a mixed effect of the physicochemical variables on BP was observed since the stations dispersed between all variables in the PLS. Despite this, the stations closest to the river mouth (i.e. stations 1, 2, 4, 5 and 8) were situated close to the ADOM components. In late summer (August), BP was consistently lower than earlier in the year, coinciding with a decrease in ADOM components and an increase in PP (Fig. 3). At this point in the season, BP was generally more influenced by pH, PP, TotP and temperature, suggesting that bacteria were more reliant on autochthonously produced carbon (Fig. 2a). In addition to lower flow rates (Table 1) and thus a less plentiful supply of ADOM during this period, the river-borne carbon may have been of lower bioavailability than in early spring [50]. These conditions could drive bacteria to a reliance on autochthonously produced DOC, switching to an autotrophic-based food web where competition for nutrients with phytoplankton could regulate the late-season (August) basal production.

### Variation of DOC Availability

**Fig. 5:**
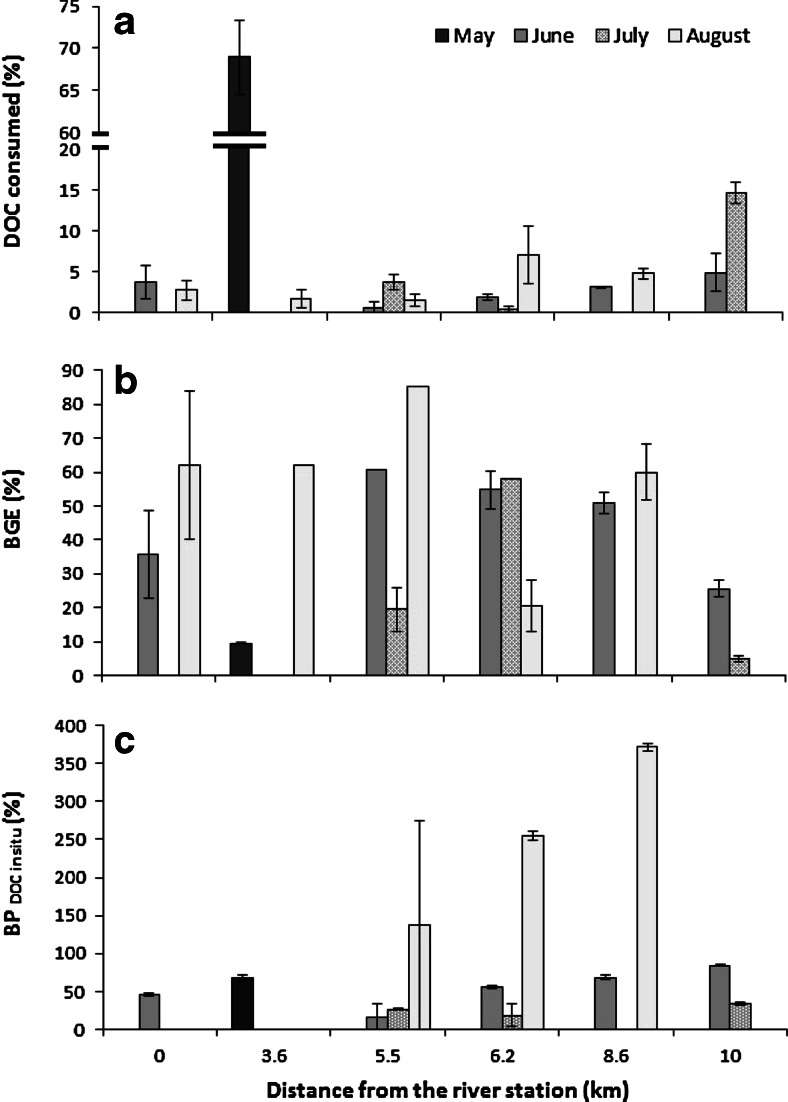
Fraction of bioavailable DOC (**a**), bacterial growth efficiency (**b**) and proportion of DOC fuelling bacterial production in situ (**c**) measured at stations along a transect from the river to 10 km offshore in the Råne Estuary during the period May–August 2011. *Error bars* are ±standard deviation

Although the highest BP was recorded during the time of peak levels of DOC and other ADOM components, the DOC availability was not consistently higher during this time of the year (compare Figs. 4a and 5a). In general, the DOC availability showed no specific spatial and temporal trend (Fig. 5a). It varied from 0 to ∼15 %, and the average value during the entire study season was ∼2 %. This is in the lower range of what has been observed further south in the northern Baltic Sea, 5–10 % [31, 44, 59], and might be explained by a higher proportion of relatively refractory ADOM in the northerly Råne Estuary compared to more southerly study locations where primary production is higher [5]. Therefore, the high bacterial production during spring was probably due to the large freshwater inflow, containing large amount of fresh ADOM. In fact, the Råne River discharge was fourfold higher at the time of the May sampling than during the sampling period in August (Table 1).

An exceptionally high DOC consumption, ∼70 %, was however recorded in the river mouth (the station at 3.6 km) during the May sampling event. This may be regarded as an extreme value but is partly supported by an earlier study, indicating that relatively high DOC degradation occurs in the shallow coastal zone of the northern Baltic Sea [19]. Furthermore, an additional study also showed higher DOC consumption in the river mouth than in the river during the spring season (data not shown). The high DOC consumption at 3.6 km from the river source station (Fig. 5a) may be a result of exposing concentrated ADOM to other biological and geochemical processes in the mixing zone between river water and seawater, priming degradation of the ADOM. Despite the relatively small salinity change, it may be enough for slight modification of the DOC compounds leading to increased bioavailability for bacterial consumption [60]. Our results are in agreement with studies showing that the DOC utilization is governed by properties of the carbon sources, which can be modified by environmental interactions [7, 8, 33, 42]. However, other aspects such as changes in community structure may further affect the bacterial function and thus ADOM consumption [24].

### Spatial and Temporal Variation of Limiting Substance

The bioassay showed that the growth-limiting substance for bacteria varied spatially and temporally (Table 3). In May–July, the inner stations were potentially carbon limited (C), while the more seaward stations tended to be NP limited. In fact, bacteria can be carbon limited even if the concentration of DOC is high, all depending on the characteristics of the DOC pool and the availability of NP nutrients [19, 29, 50]. Since labile DOC was consumed along the river-sea gradient, high-quality available DOC may not have reached the seaward stations, where bacteria may have been more reliant on phytoplankton-produced DOC, increasing competition for nutrients between bacteria and phytoplankton, as is common in open sea ecosystems [40]. In line with this, most of the stations (70 %) were NP limited in August, when the PP was highest, probably boosted, particularly at seaward stations, by the better light climate due to lower ADOC inputs.

**Table 3:** Nitrogen/phosphorous (NP) limitation of bacterial growth, analysed by the bioassay

Distance (km)	Station	Limiting ADOC bacterial consumption			
		May	June	July	August
0	1	C_decrease_	C_decrease_	C_decrease_	NP_limited_*
3.6	2	C_decrease_	C_decrease_	C_decrease_	C_decrease_
5.5	6	C_decrease_	C_decrease_	NP_limited_*	C_decrease_
6.2	10	C_decrease_	NP_limited_*	C_decrease_	NP_limited_*
8.6	17	C_decrease_	NP_limited_*	C_decrease_	NP_limited_**
10	19	NP_limited_*	NP_limited_*	NP_limited_	NP_limited_**

The NP limitation of DOC bacterial consumption was determined by comparing BP using linear mixed model comparison between cultures with and without nutrient addition. The data were examined using ANOVA, with levels of significance indicated by: *p < 0.05, **p < 0.01, ***p < 0.001

TotN concentrations were generally higher in May than during later sampling events (Fig. 4b). Throughout the study season, the TotN concentrations were higher in the river than at the seaward stations, confirming that the river water was the major nitrogen source (Fig. 4b). The TotP concentration was low during the whole productive season, while no significant trend was observed along the gradient except in June, when TotP was generally lower at seaward stations (Fig. 4c). Wikner and Andersson [58] showed in a long time series study in the coastal Bothnian Sea that discharge of total C and N was strongly correlated with riverine flows, while TotP was not, as we also observed in the Råne Estuary. Thus, it is likely that the river was not the main supplier of P (Fig. 4c). A specific limiting nutrient could not be directly determined in the bioassay since a mix of inorganic NP was utilized. However, we find it likely that P was the most limiting factor, as has been reported for this sea area in earlier studies [3, 61].

### Contribution of ADOC to Bacterial Production

The average bacterial growth efficiency (BGE) was ∼40 %, which is comparable to previous studies in the Baltic Sea, and in other waters receiving both autochthonous and allochthonous carbon [9, 20]. However, in our study, the BGE tended to be negatively related to the DOC consumed (Fig. 5a, b). This may be explained by a combination of high quantity and low quality DOC. We did not measure the quality of the DOC; however, if the DOC contained high molecular-weight (HMW) compounds, a large amount of carbon would be respired [11]. Considering that 80 % of the ADOM in the Bothnian Bay originates from the terrestrial system [2], a large part of the carbon could be lost by respiration thus influencing CO_2_ fluxes [7]. This would further explain the uncoupling or the negative relationship between BGE and DOC consumption [1].

The estimated potential ADOC support for in situ bacterial production (BP_DOC in situ_) showed large temporal and spatial variations (Fig. 5c), ranging from 0 % to values above 100 %. The highest values were recorded during late summer, in August, when values of 130–370 % were observed at some of the study locations. This may appear un-realistically high but may partly be explained by a much higher predation-pressure on the bacteria in August than in May. The carbon biomass concentration of heterotrophic nanoflagellates (HNF), the main predators of bacteria [27], was significantly higher in August than in May, on average 1328 and 303 μg C m^−3^ respectively (Kruskal-Wallis test_(*df* = 1)_, *χ*^2^ = 8.2, **p < 0.01). The predation-pressure on bacteria by heterotrophic nanoflagellates, calculated as HNF/BP, was ∼20-fold higher in August than in May. This may have contributed to the relatively low BP in situ recorded in August (Fig. 3a). In addition to the high predation-pressure on bacteria, phytoplankton production was higher than BP in August (Fig. 3a), which could have enriched the DOC with autochthonous DOC. Consequently, the BGE increased (Fig. 5b) in the bioassay, resulting in high estimated potential ADOC support in situ during August (Fig. 5c). However, overall, the ADOC support of in situ bacterial production was, on average, 60 % during the studied season, suggesting that ADOC represents an important carbon and energy source for bacterial production in this estuary (Fig. 2 and Table 2).

These results, based on the bioassay together with the multivariate analysis of in situ measurements, strongly support the suggestion that ADOC was a significant driver of bacterial production. We demonstrate that the studied subarctic estuary is regulated in a similar way to humic and unproductive subarctic lakes, where the pelagic production is regulated by the concentration of ADOM [6, 37]. Ask et al. [6] showed that ADOM decreased autotrophic production in humic lakes, turning the ecosystem toward heterotrophic-dominated basal production. Furthermore, Karlsson et al. [36] confirmed that ADOM decreases the intermediate and top consumers’ biomass across a range of different lake conditions. Our study reports similar effects of ADOM on the basal production in coastal waters of the northern Baltic Sea, suggesting that ADOM could subsequently decrease energy transfer to mesozooplankton, a factor that is of significance in coastal and estuarine zones that are of vital importance to higher organisms such as fish. The results of this study are also important for the understanding of how climate change will affect coastal areas in the northern Europe. Changes in climate are predicted to result in a 15 to 20 % increase in runoff to the Baltic Sea by 2100 [47], with the largest changes expected in the northernmost part (the Gulf of Bothnia) due to a large number of in-flowing rivers [25]. Moreover, increases in ADOM discharges are expected as these rivers drain vast and largely forested catchments, further reducing ecosystem production in the northern Baltic Sea [52]. It is clear that pelagic production is highly influenced by ADOM in coastal areas in the northern Baltic Sea; however, further investigations are needed to clarify the knock-on effect of ADOM increases on organisms of higher trophic levels.

## Conclusion

In conclusion, our results show that the BP in the studied subarctic estuary is governed by the availability and concentration of ADOM. It is also clear that the nutrient status and bioavailability are of vital importance when defining bacterial carbon utilization, offering important pointers that may control these processes, even under apparently plentiful DOC concentrations. Furthermore, spatial and seasonal environmental variations as well as changes in the function of different bacterial communities may influence the DOC utilization. Prevailing physicochemical conditions, which are influenced by other environmental factors (e.g. flow rates), play a significant role in controlling BP. The dominance of BP in total pelagic production indicates that bacteria support higher trophic levels in this ecosystem. Climate change scenarios show that precipitation and river discharge will increase causing higher inflow of ADOM to the coastal area, which in turn will decrease phytoplankton production and further promote the BP. In this scenario, the dominance of heterotrophic pelagic production could increase the ADOM consumption, potentially increase bacterial respiration levels and thus release more CO_2_ from coastal estuaries in the Baltic Sea. Food webs would probably be elongated and become less efficient, decreasing productivity at higher trophic levels and decreasing the total productivity of such coastal ecosystems.

## Electronic supplementary material

Below is the link to the electronic supplementary material.

ESM 1 (DOCX 13 kb)
