# Supplementary material for: Allochthonous Carbon—a Major Driver of Bacterioplankton Production in the Subarctic Northern Baltic Sea
Source: Microb Ecol. 2015 Dec 17;71:789–801. doi: 10.1007/s00248-015-0714-4 (PMC4823372; doi:10.1007/s00248-015-0714-4)
Supplement: Supplementary file 1 — (DOCX 13 kb) [file 248_2015_714_MOESM1_ESM.docx]

SUPLEMENTARY INFORMATION:

**S. Table 1:** Distance from the river (station 1), coordinates and depth of the studied stations at Råne estuary.

| **Station** | **Distance (km)** | **Latitude** | **Longitude** | **Depth (m)** |
| --- | --- | --- | --- | --- |
| **1** | 0 | 65° 51.3383 N | 22° 16.8642 E | 0.6 |
| **2** | 3.6 | 65° 50.1147 N | 22° 20.5801 E | 1.8 |
| **3** | 4.4 | 65° 50.362 N | 22° 22.197 E | 1.1 |
| **4** | 5.2 | 65° 49.494 N | 22° 21.934 E | 1 |
| **5** | 5.2 | 65° 49.7041 N | 22° 22.3374 E | 2 |
| **6** | 5.5 | 65° 49.9328 N | 22° 23.2160 E | 3.4 |
| **7** | 6.0 | 65° 50.0932 N | 22° 24.1293 E | 3.5 |
| **8** | 5.4 | 65° 49.2024 N | 22° 21.6145 E | 1.3 |
| **9** | 5.7 | 65° 49.3427 N | 22° 22.6109 E | 3.2 |
| **10** | 6.2 | 65° 49.4899 N | 22° 23.6573 E | 1.8 |
| **11** | 7.1 | 65° 49.7073 N | 22° 25.3022 E | 4.7 |
| **12** | 6.6 | 65° 48.5482 N | 22° 22.2100 E | 6 |
| **13** | 7.0 | 65° 48.6405 N | 22° 23.1902 E | 4.8 |
| **14** | 7.4 | 65° 48.7537 N | 22° 24.3034 E | 4 |
| **15** | 8.2 | 65° 48.961 N | 22° 25.905 E | 5.9 |
| **16** | 7.7 | 65° 47.9415 N | 22° 22.7209 E | 10.7 |
| **17** | 8.6 | 65° 47.9752 N | 22° 24.5837 E | 4.7 |
| **18** | 9.5 | 65° 47.9324 N | 22° 26.1150 E | 5 |
| **19** | 10.1 | 65° 48.0112 N | 22° 27.2783 E | 6.6 |
